# Sexual Transmission of Zika Virus and Persistence in Semen, New Zealand, 2016

**DOI:** 10.3201/eid2210.160951

**Published:** 2016-10

**Authors:** Jay Harrower, Tomasz Kiedrzynski, Simon Baker, Arlo Upton, Fahimeh Rahnama, Jill Sherwood, Q. Sue Huang, Angela Todd, David Pulford

**Affiliations:** Auckland Regional Public Health Service, Auckland, New Zealand (J. Harrower, S. Baker);; Ministry of Health, Wellington, New Zealand (T. Kiedrzynski);; Labtests and Northland Pathology Laboratory, Whangarei, New Zealand (A. Upton);; Auckland District Health Board, Auckland (F. Rahnama);; Institute of Environmental Science and Research Limited, Wallaceville, New Zealand (J. Sherwood, Q. Sue Huang, A. Todd);; Ministry for Primary Industries, Wallaceville (D. Pulford)

**Keywords:** Zika, Zika virus, sexual transmission, semen, arbovirus, arboviral disease, flavivirus, viruses, New Zealand

**To the Editor:** Zika virus infection is an emerging arboviral disease linked to an increased risk for severe neurologic outcomes and devastating adverse fetal complications, including pregnancy loss and congenital microcephaly (*1*). Most Zika virus infections result from bites from mosquito vectors; however, increasing evidence indicates that Zika virus infection can be sexually transmitted (*1*–*3*). Probable sexual transmission from male to female (*3*–*5*) and male to male (*6*) have been reported, and transmission through vaginal, anal (*6*), and oral (*5*) sexual intercourse has been implicated. Studies also have demonstrated the presence of Zika virus RNA in semen 2 weeks after symptom onset, with viral loads ≈100,000 times greater than those detected concurrently in serum (*7*), and Zika virus RNA has been detected in semen while being undetectable in serum (*2*,*5*,*8*). The presence of Zika virus RNA in semen up to 62 days after symptom onset of Zika virus infection has been reported (*8*). Here we report a case of locally acquired Zika virus infection that was almost certainly the result of sexual transmission and our findings indicating the duration of Zika virus RNA persistence in semen.

A 51-year-old man (patient 1) who regularly traveled to Samoa, a Pacific island with known Zika virus transmission, returned to New Zealand in late January 2016. He reported having been sexually abstinent while overseas. After 1 week he experienced onset of fever, rash, arthralgia, and ankle edema. Aware of the symptoms of mosquito-borne illnesses prevalent in his region of travel, he assumed he had a mild case of chikungunya and sought no medical attention.

A 53-year-old woman (patient 2) who was the sexual partner of patient 1 experienced onset of sore throat, fever, arthralgia, and rash 17 days after her partner’s return to New Zealand (10 days after he had onset of symptoms). She had had no recent overseas travel, had no blood transfusions, and was unaware of having any mosquito bites. Four days after symptom onset, she visited a general practitioner, whose investigations revealed mild neutropenia, normal liver and renal function, no evidence of streptococcal throat infection, and a demonstrated immunity to measles.

The rash subsequently spread, and small joint arthralgia worsened. The patient returned to the general practitioner 9 days after symptom onset. Disclosing her partner’s travel history and symptoms and reporting having unprotected sexual intercourse with him after his return to New Zealand (before and during the time he was symptomatic), she enquired about the possibility of Zika virus infection. After discussing the matter with a clinical microbiologist and securing the consent of the woman and her partner, the general practitioner ordered tests for arboviruses common in the male partner’s region of travel. 

All laboratory tests for chikungunya and dengue viruses were negative for both patients. Testing for Zika virus RNA was performed by using real-time reverse transcription PCR (rRT-PCR) in accordance with published methods (Table) (*9*). Serum from patient 2 tested negative for Zika virus RNA when first tested on day 9 after symptom onset, although subsequent retrospective testing on the serum sample collected on day 4 returned a positive result. A urine sample collected on day 9 was positive for Zika virus RNA. These tests were repeated 3 days later and returned identical results. Testing of serum collected on day 9 detected Zika IgM and IgG (IgG titer >1:640), and similar results were obtained on serum collected on day 12.

**Table:** Timeline and results of rRT-PCR testing on specimens from 2 patients with suspected Zika virus infection, New Zealand, 2016*

Patient no. (age, y/sex)	Days after initial return of patient 1 to New Zealand	Days after symptom onset	Specimen type	Result of rRT-PCR for Zika virus
Patient 1 (51/M)	26	19	Serum	–
28	21	Urine	–
28	21	Serum	–
30	23	Semen	+
42	35	Semen	+
83	76	Semen	+
106	99	Semen	–
124	117	Semen	–

Patient 2 (53/F)	21†	4†	Serum	+
26	9	Serum	–
26	9	Urine	+
29	12	Serum	–
29	12	Urine	+

*rRT-PCR, real-time reverse transcription PCR; +, positive; –, negative. †Performed retrospectively on stored serum after observation of positive results obtained on urine samples collected on days 9 and 12 after symptom onset.

Laboratory investigations for Zika virus RNA in patient 1 demonstrated negative rRT-PCR results for serum (first tested 19 days after symptom onset) and for urine (first tested 21 days after symptom onset). Serologic testing performed on day 21 detected Zika IgM and IgG (IgG titer 1:320). Semen collected on day 23 tested positive for Zika virus RNA (cycle threshold [C_t_] 25). Semen collected on days 35 and 76 also tested positive (C_t_ values 29 and 35, respectively; duplicate testing on the sample from day 76 performed at the national arbovirus reference laboratory indicated a C_t_ value of 35.82). Semen samples collected on days 99 and 117 tested negative for Zika virus RNA. Attempts at virus isolation from the semen sample collected on day 23 failed to cultivate infectious particles.

It is very unlikely that transmission of Zika virus infection to patient 2 occurred through a mosquito bite. Although occasional interceptions of exotic mosquito species have occurred at international ports of entry into New Zealand, neither of the *Aedes* species of mosquito capable of transmitting Zika virus infection is established in the country (*10*). This case report and results of research into the duration of infectivity of Zika virus in semen can inform the evolving guidelines concerning the recommended duration of abstinence from sexual intercourse and the practice of barrier protection methods to prevent sexual transmission of Zika virus infection.
